# TORC1 specifically inhibits microautophagy through ESCRT-0

**DOI:** 10.1007/s00294-019-00982-y

**Published:** 2019-04-30

**Authors:** Riko Hatakeyama, Claudio De Virgilio

**Affiliations:** Department of Biology, University of Fribourg, 1700 Fribourg, Switzerland

**Keywords:** Target of rapamycin complex 1 (TORC1), Endosomal sorting complex required for transport (ESCRT), Multivesicular body, Microautophagy, Vps27

## Abstract

Nutrient starvation induces the degradation of specific plasma membrane proteins through the multivesicular body (MVB) sorting pathway and of vacuolar membrane proteins through microautophagy. Both of these processes require the gateway protein Vps27, which recognizes ubiquitinated cargo proteins at phosphatidylinositol 3-phosphate-rich membranes as part of a heterodimeric complex coined endosomal sorting complex required for transport 0. The target of rapamycin complex 1 (TORC1), a nutrient-activated central regulator of cell growth, directly phosphorylates Vps27 to antagonize its function in microautophagy, but whether this also serves to restrain MVB sorting at endosomes is still an open question. Here, we show that TORC1 inhibits both the MVB pathway-driven turnover of the plasma membrane-resident high-affinity methionine permease Mup1 and the inositol transporter Itr1 and the microautophagy-dependent degradation of the vacuolar membrane-associated v-ATPase subunit Vph1. Using a Vps27^7D^ variant that mimics the TORC1-phosphorylated state of Vps27, we further show that cargo sorting of Vph1 at the vacuolar membrane, but not of Mup1 and Itr1 at endosomes, is sensitive to the TORC1-controlled modifications of Vps27. Thus, TORC1 specifically modulates microautophagy through phosphorylation of Vps27, but controls MVB sorting through alternative mechanisms.

## Introduction

Living cells control growth in a homeostatic manner by tightly coupling protein synthesis and catabolism to the availability of nutrients. Upon nutrient starvation, cytosolic proteins are degraded through macroautophagy, while specific plasma membrane- and lysosomal membrane-resident proteins are catabolized through the multivesicular body (MVB) sorting pathway and microautophagy, respectively (Müller et al. 2015; Oku et al. 2017; Zhu et al. 2017). Macroautophagy requires a defined set of autophagy-related (Atg) proteins (Mizushima et al. 2011; Wen and Klionsky 2016) and is mechanistically different from MVB sorting, which relies on the endosomal sorting complex required for transport (ESCRT) machinery (Henne et al. 2011). Interestingly, recent studies have demonstrated that ESCRT also drives microautophagy at lysosomal (i.e., yeast vacuolar) membranes, although ESCRT-independent subtypes of microautophagy may also exist (Oku and Sakai 2018). In both processes, MVB sorting at endosomal membranes and ESCRT-driven microautophagy at vacuolar membranes, Vps27 forms a heterodimer with Hse1 (named ESCRT-0) that initiates the sorting of membrane-resident and associated proteins following their posttranslational ubiquitination. Accordingly, cargo recognition by ESCRT-0 is followed by invagination of the cargo-containing membrane and formation of intraluminal vesicles that are directly degraded within the vacuole (during microautophagy) or following the fusion of endosome-derived MVBs with the vacuole (during the ultimate step of the MVB pathway). How nutrients control these processes has largely remained elusive.

The target of rapamycin complex 1 (TORC1) is a central integrator of nutritional information that regulates various anabolic and catabolic processes to ensure cellular homeostasis in eukaryotic cells (Albert and Hall 2015; Eltschinger and Loewith 2016; Saxton and Sabatini 2017). In yeast, TORC1 inhibits macroautophagy via direct phosphorylation of Atg13, which reduces its affinity for and promotes dissociation from Atg1, a protein kinase that centrally induces macroautophagy when in complex with Atg13 (Kamada et al. 2010). TORC1 also controls the MVB pathway-driven degradation of plasma membrane proteins (Dauner et al. 2017; Dobzinski et al. 2015; Jones et al. 2012; MacGurn et al. 2011), and conceivably also the microautophagic turnover of vacuolar membrane proteins (Oku et al. 2017; Rahman et al. 2018; Zhu et al. 2017), although the underlying mechanisms have remained incompletely understood. In this context, we recently identified two spatially and functionally distinct pools of TORC1 that reside on the limiting membranes of vacuoles and some endosomes that we coined signaling endosomes (Hatakeyama and De Virgilio 2019; Hatakeyama et al. 2019). Interestingly, vacuolar TORC1 plays a prominent role in regulating protein synthesis via its proximal effector Sch9 (Ejzykowicz et al. 2017; Hatakeyama et al. 2019; Jin et al. 2014; Urban et al. 2007). Endosomal TORC1, in contrast, phosphorylates Atg13 to antagonize initiation of macroautophagy, which occurs at perivacuolar foci called preautophagosomal structures that are in close proximity to endosomal membranes (Hatakeyama et al. 2019; Suzuki et al. 2013). Endosomal TORC1 is further specifically commissioned to phosphorylate the bulk of Vps27 (at 7 Ser/Thr residues) and thereby antagonize the ESCRT-driven microautophagic degradation of the vacuolar membrane protein phosphatase Pho8. Whether the TORC1-mediated phosphorylation of Vps27 also affects cargo sorting through the MVB pathway, however, is currently not known.

Here, we show that TORC1 inhibits sorting and degradation of both specific plasma membrane proteins (via the MVB pathway) and of the vacuolar membrane protein Vph1 (via microautophagy). Using a Vps27^7D^ variant that mimics the TORC1-triggered phosphorylated state of Vps27, we further show that cargo sorting during microautophagy, but not MVB sorting, is sensitive to the TORC1-controlled modifications of Vps27. Thus, TORC1 appears to specifically tune microautophagy, but not MVB sorting, through phosphorylation of Vps27.

## Materials and methods

### Yeast strains, plasmids, and growth conditions

*Saccharomyces cerevisiae* strains used in this study, all derived from BY4741/2 (Brachmann et al. 1998), are RKH119 (MATa, *vps27∆*::*KanMX*, *his3∆1*, *leu2∆0*, *ura3∆0*), RKH495 ([RKH119] *VPH1*-*EGFP::HIS3*), RKH463 ([RKH119] *MUP1*-*EGFP::HIS3*), RKH483 ([RKH119] *ITR1*-*GFP::HIS3 met15∆0*), and RKH360 ([RKH119] *URA3::P*_*PHO5*_-*GFP*-*CPS1 met15∆0*). The plasmids used in this study are: pRS415 (CEN, ARS, *LEU2*) and pRS416 (CEN, ARS, *URA3*) (Sikorski and Hieter 1989); p1379 (CEN, ARS, *URA3, MET15*), p3505 (CEN, ARS, *LEU2, VPS27*), and p3550 (CEN, ARS, *LEU2, VPS27*^*S155D/S157D/T159D/S274D/S277D/S279D/S280D*^) (Hatakeyama et al. 2019); and p1770 (CEN, ARS, *HIS3, MET15*), p3628 (CEN, ARS, *URA3, VPS27*) and p3629 (CEN, ARS, *URA3, VPS27*^*S155D/S157D/T159D/S274D/S277D/S279D/S280D*^) (this study). Yeast strains were made prototrophic by complementing auxotrophic markers with empty vector plasmids and grown to exponential growth phase in synthetic dextrose medium (0.17% yeast nitrogen base, 0.5% ammonium sulfate, and 2% glucose) at 30 °C. In Fig. 2a, b, leucine (14.8 mg l^−1^ final concentration) was supplemented to the medium to support the expression of Mup1-GFP.

### Fluorescence microscopy

Images of live fluorescent cells were captured with an inverted spinning disk confocal microscope (VisiScope CSU-W1, Puchheim, Germany) that is equipped with a scientific grade 4.2 sCMOS camera and a 100 × 1.3 NA oil immersion Nikon CFI series objective (Egg, Switzerland), and processed using ImageJ software. For each strain and condition, at least 60 cells were recorded and the pictures show representative cells in each case.

### Cell lysate preparation and immunoblot analyses

Cells were treated with 6.7% w/v trichloroacetic acid (final concentration) for at least 10 min on ice, pelleted, washed with ice-cold 70% ethanol, dried, dissolved in urea buffer [50 mM Tris–HCl (pH 7.5), 5 mM EDTA, 6 M urea, 1% SDS, 0.4 mM Pefabloc (Sigma-Aldrich), and PhosSTOP (Roche, one tablet per 10 ml)], and disrupted with glass beads using a Precellys homogenizer. After being heated at 65 °C for 10 min twice, with the second heating performed in Laemmli SDS sample buffer, samples were subjected to SDS-PAGE and transferred to nitrocellulose membranes. Subsequent to blocking with blocking buffer (1% bovine serum albumin and 5% milk powder in phosphate-buffered saline), membranes were immunoblotted with the following primary antibodies diluted in the blocking buffer: mouse anti-GFP (Roche, product number 118144600001; 1:1000 dilution), rabbit anti-Vps27 (lab stock; 1:1000 dilution) or rabbit anti-Adh1 (Calbiochem, product number 126745; 1:200,000 dilution). After washing with Tris-buffered saline containing 0.3% Tween 20, membranes were incubated with mouse (BIO-RAD, product number 170-6516; 1:3000 dilution) or rabbit (BIO-RAD, product number; 170-6515; 1:3000 dilution) secondary antibodies conjugated with horseradish peroxidase, washed again and developed with ECL (GE Healthcare). All immunoblot analyses were carried out in triplicates (biological replicates) of which one representative blot is shown in each case.

## Results

### TORC1 controls microautophagic degradation of Vph1 via ESCRT-0

**Fig. 1:**
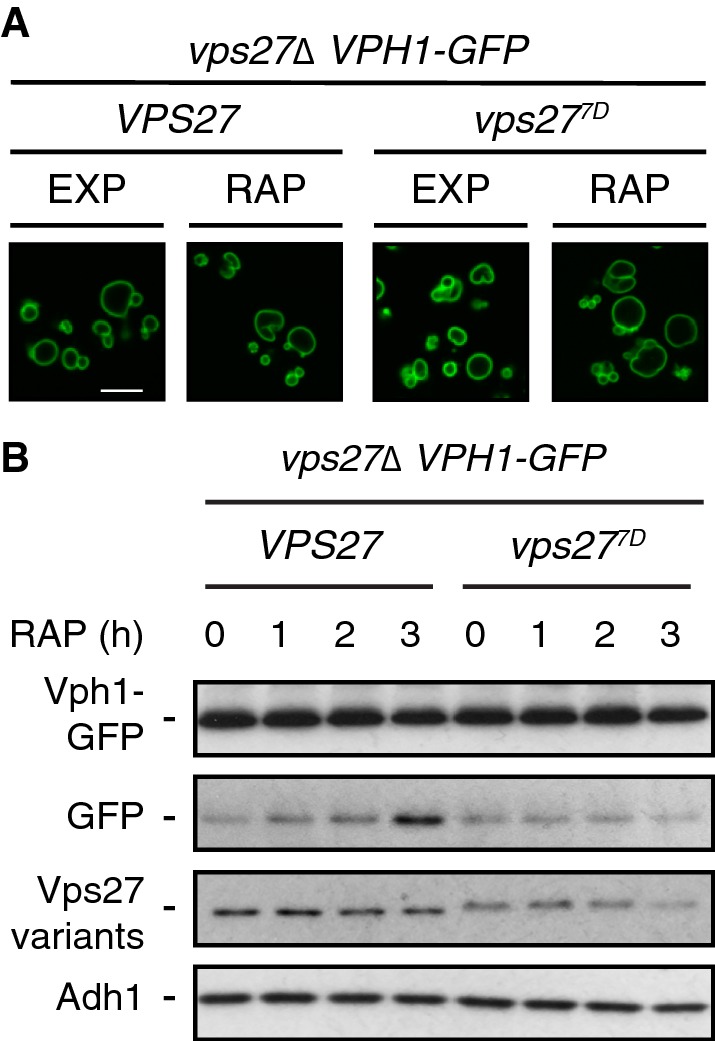
TORC1 controls microautophagic degradation of Vph1 via Vps27. **a** Vph1-GFP is properly sorted to the vacuolar membrane in *vps27*^*7D*^ cells. Cells (*vps27∆*) expressing genomically-tagged Vph1-GFP and plasmid-encoded Vps27 or Vps27^7D^ were grown to exponential growth phase, treated (RAP) or not (EXP) for 3 h with 200 ng ml^−1^ rapamycin, and visualized by fluorescence microscopy. Scale bar, 5 µm (white). **b** Vps27^7D^-expressing cells are defective in microautophagic degradation of Vph1-GFP. The strains used in **a** were grown to exponential growth phase, treated with 200 ng ml^−1^ rapamycin for the times indicated, and subjected to immunoblot analyses (using anti-GFP antibodies) to measure the levels of GFP cleavage from Vph1-GFP. Notably, TORC1 inactivation also triggered the slow degradation of Vps27 and Vps27^7D^ over time. This effect is likely caused by combined degradation of the Vps27 variants through the MVB pathway [as it is partially blocked in the absence of the vacuolar protease Pep4 (Hatakeyama et al. 2019)] and through proteasome-mediated degradation (Dobzinski et al. 2015). A minor fraction of Vps27, but likely not of Vp27^7D^, may be routed via microautophagy for degradation in the vacuole (Hatakeyama et al. 2019). The expression levels of Vps27 and Vps27^7D^ were analyzed using anti-Vps27 antibodies. Adh1 levels, probed with anti-Adh1 antibodies, served as loading controls

We have previously described that rapamycin-treated cells expressing the phosphomimetic Vps27^7D^ variant were significantly impaired in microautophagic degradation of the vacuolar membrane-resident phosphatase Pho8 (Hatakeyama et al. 2019). To corroborate and extend these findings, we also examined the fate of an additional vacuolar membrane protein, namely the v-ATPase subunit Vph1 (Forgac 2007), which has recently been found to undergo Vps27-dependent microautophagic degradation in rapamycin-treated cells (Rahman et al. 2018). A caveat in the latter study, however, is that Vph1 travels through endosomes to reach the vacuole and that MVB sorting mutants (including the ones with defects in ESCRT subunits) accumulate Vph1 in specific perivacuolar, endosomal membrane-derived structures that are collectively coined the class E compartment (Raymond et al. 1992; Rieder et al. 1996; Rothman 1989). Consequently, Vph1-GFP cannot reach the vacuolar membrane in *vps27∆* cells, which is why Vph1-GFP cleavage per se is unlikely to report microautophagic activity in these cells (Oku et al. 2017; Rahman et al. 2018). In contrast, our observation that both Vps27 and Vps27^7D^ functioned properly in sorting Vph1 to the vacuolar membrane (Fig. 1a), provided us with a unique opportunity to assess the role of the Vps27^7D^ allele in microautophagic degradation of Vph1-GFP. Gratifyingly, and in line with our previous data on GFP-Pho8 degradation (Hatakeyama et al. 2019), rapamycin treatment induced the degradation of Vph1-GFP (visualized by the accumulation of the stable, free GFP moiety) in Vps27- but not in Vps27^7D^-expressing cells (Fig. 1b). Thus, TORC1 regulates microautophagy of Pho8 and of Vph1 via phosphorylation of Vps27.

### TORC1 does not modulate MVB sorting of Mup1 and Itr1 through Vps27 phosphorylation

**Fig. 2:**
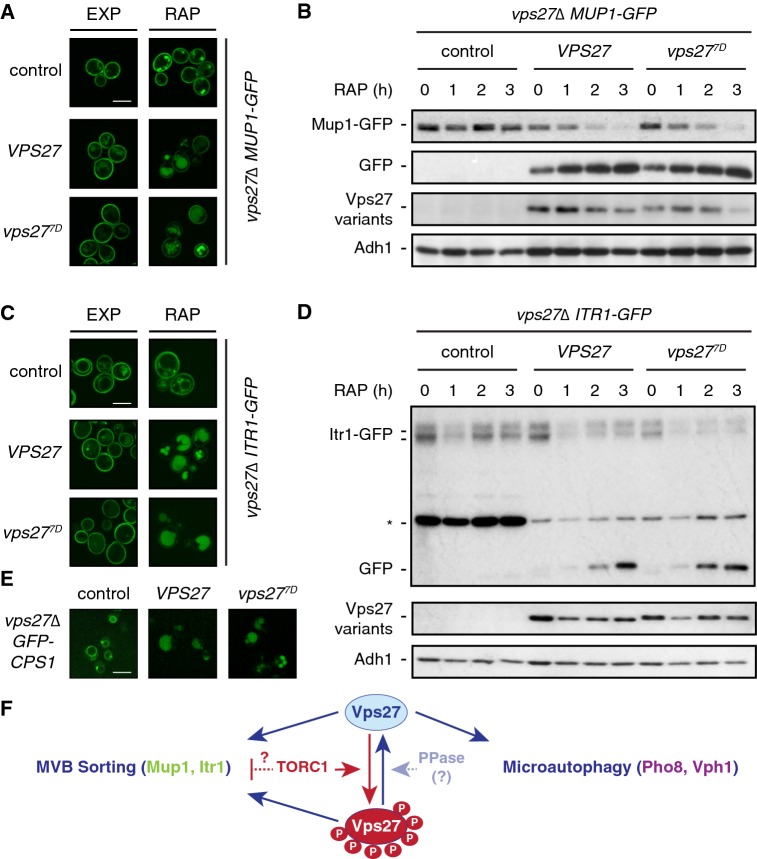
TORC1-mediated phosphorylation of Vps27 does not affect MVB sorting. **a** Mup1-GFP is normally sorted to the vacuolar lumen in rapamycin-treated, Vps27^7D^-expressing cells. Cells (*vps27∆*) expressing genomically-tagged Mup1-GFP together with (or without; control) plasmid-encoded Vps27 or Vps27^7D^ were grown to exponential growth phase, treated (RAP) or not (EXP) for 3 h with 200 ng ml^−1^ rapamycin, and visualized by fluorescence microscopy. Notably, loss of Vps27 (control) caused Mup1-GFP to remain at the plasma membrane and accumulate within presumably the class E compartment where it is protected from degradation in rapamycin-treated cells. Scale bar, 5 µm (white). **b** TORC1 inhibition normally induces vacuolar degradation of Mup1-GFP in *vps27*^*7D*^ cells. The strains used in **a** were analyzed as in Fig. 1b. **c** Itr1-GFP is normally sorted to the vacuolar lumen in rapamycin-treated, Vps27^7D^-expressing cells. Cells (*vps27∆*) expressing genomically-tagged Itr1-GFP together with (or without; control) plasmid-encoded Vps27 or Vps27^7D^ were grown to exponential growth phase, treated (RAP) or not (EXP) for 3 h with 200 ng ml^−1^ rapamycin, and visualized by fluorescence microscopy. Notably, loss of Vps27 (control) caused Itr1-GFP to be partially and constitutively missorted to the vacuolar membrane. Scale bar, 5 µm (white). **d** TORC1 inhibition normally induces vacuolar degradation of Itr1-GFP in *vps27*^*7D*^ cells. The strains used in **c** were analyzed as in Fig. 1b. Notably, extracts of *vps27∆* control cells contain no free GFP moieties, but exhibit constitutively higher levels of a 45-kDa Itr1-GFP cleavage product (denoted with an asterisk) than Vps27 and Vps27^7D^-expressing cells, which we presume is causally related to the observed missorting of Itr1-GFP in these cells. **e** GFP-Cps1 is normally sorted to the vacuolar lumen in exponentially growing *vps27*^*7D*^ cells. Cells (*vps27∆*) expressing genomically-tagged GFP-Cps1 together with (or without; control) plasmid-encoded Vps27 or Vps27^7D^ were grown to exponential growth phase and visualized by fluorescence microscopy. Scale bar, 5 µm (white). **f** Microautophagy, but not MVB sorting, is sensitive to TORC1-mediated phosphorylation of Vps27. Arrows and bars denote positive and negative regulatory mechanisms, respectively. PPase and P denote a still elusive protein phosphatase and phosphorylated residues, respectively. For details, see text

Nutrient starvation and/or inactivation of TORC1 is known to promote the endocytosis, sorting through the MVB, and ultimate degradation within the vacuolar lumen of multiple cell surface proteins (Lang et al. 2014; Müller et al. 2015; Schmidt et al. 1998). To study a role of TORC1-mediated Vps27 phosphorylation in MVB sorting, we set out to identify plasma membrane proteins that were subjected to Vps27-dependent vacuolar degradation upon treatment of cells with rapamycin. One good candidate protein, we assumed, was the plasma membrane-resident high-affinity methionine permease Mup1, which is specifically degraded through the MVB pathway when cells are starved for nutrients (Müller et al. 2015). Indeed, Mup1 fulfilled our criteria, as Mup1-GFP localized at the plasma membrane in the presence and absence of Vps27 in exponentially growing cells, but disappeared from the plasma membrane and accumulated within vacuoles in a Vps27-dependent manner when cells were treated with rapamycin (Fig. 2a). In line with these cell biological data, immunoblot analyses confirmed that rapamycin treatment triggered the Vps27-dependent degradation of Mup1-GFP (and a parallel accumulation of free, stable GFP; Fig. 2b). Importantly, Mup1-GFP localization, sorting, and degradation were indistinguishable between cells expressing Vps27 and the ones expressing Vps27^7D^ (Fig. 2a, b). To corroborate these data, we also studied the plasma membrane inositol transporter Itr1, which, like Mup1, has been found to undergo nutrient starvation-induced degradation (Müller et al. 2015). Accordingly, Itr1-GFP localized at the plasma membrane in exponentially growing cells, but disappeared from the plasma membrane and accumulated within vacuoles similarly in both Vps27 and Vps27^7D^-expressing cells (Fig. 2c). Again, as with Mup1-GFP, immunoblot analyses confirmed that rapamycin treatment triggered the degradation of Itr1-GFP (and induced the accumulation of free, stable GFP; Fig. 2d) indistinguishably between Vps27- and Vps27^7D^-expressing cells (Fig. 2d). Thus, TORC1 inhibits MVB sorting of Mup1-GFP and Itr1-GFP via mechanisms that do not depend on phosphorylation of Vps27.

### TORC1-mediated phosphorylation of Vps27 does not affect steady state MVB sorting

Our analyses of Vph1-GFP indicated that Vps27^7D^ functioned normally in MVB sorting in exponentially growing cells (Fig. 1a). To further substantiate this notion, we also examined the localization of the vacuolar peptidase Cps1, which is continuously delivered to the vacuolar lumen through ESCRT-driven MVB sorting in exponentially growing cells (Brune et al. 2019; Odorizzi et al. 1998). GFP-Cps1 accumulated in the class E compartment and to some extent on the vacuolar surface in exponentially growing *vps27∆* cells as previously described (Mageswaran et al. 2015; Odorizzi et al. 1998), but normally reached the vacuolar lumen in both Vps27- and Vps27^7D^-expressing cells (Fig. 2e). Thus, the steady state flux through the MVB pathway appears not to be restrained by TORC1-mediated phosphorylation of Vps27.

## Discussion

The data presented here not only corroborate our recent discovery according to which TORC1 controls microautophagy through phosphorylation of Vps27, but also indicate that the respective regulatory mechanism does not modulate the flux through the MVB pathway. The underlying mechanistic details of this differential importance of Vps27 phosphorylation for microautophagy and MVB sorting is currently elusive, but may be related to the observation that Vps27 is mainly targeted by TORC1 on endosomal membranes, which are very rich in phosphatidylinositol 3-phosphate (PI3P) when compared to vacuolar membranes (Marat and Haucke 2016). Accordingly, we speculate that TORC1-mediated phosphorylation of Vps27 may reduce its capacity to act as a coincidence detection module that binds ubiquitinated cargo on membranes through both its ubiquitin-interacting and PI3P-binding domains (Henne et al. 2011). In such a scenario, Vps27 may be able to dock onto ubiquitinated cargo on PI3P-rich endosomes, but may be compromised to do so on vacuolar membranes as long as it is kept in its phosphorylated state by endosomal TORC1. The binding and dissociation constants between Vps27 and its ligands combined would then define the fraction of Vps27 that is released from endosomes to probe ubiquitinated cargoes on the vacuolar membrane. This model could be addressed in the future by studying the respective biochemical parameters in vitro (using purified variants of Vps27 and differently decorated liposomes) and microscopic imaging of Vps27 mobility in vivo [using for instance tandem fluorescent protein timers as described (Khmelinskii et al. 2012)].

Although TORC1 does not apparently modulate MVB sorting through Vps27, it antagonizes the endocytosis and MVB pathway-driven degradation of certain plasma membrane permeases [e.g., Mup1 and Itr1 (studied here), or the high-affinity tryptophan permease Tat2 (Beck et al. 1999)] via alternative means. It may do so in part by inhibiting the effector protein kinase Npr1, which controls the ubiquitination state, and consequently the trafficking through the endocytic pathway, of various plasma membrane proteins via mechanisms that implicate arrestin-related ubiquitin ligase adaptors and their associated ubiquitin ligases (Beck et al. 1999; Jones et al. 2012; MacGurn et al. 2011; Merhi and André 2012). The respective mechanisms remain largely to be elucidated, but TORC1 can regulate the access of plasma membrane proteins to the endocytic pathway rather specifically and, depending on the protein, in opposite ways. In this context, it is worth noting that both Mup1 and Itr1 also undergo substrate-induced endocytosis (Lai et al. 1995; Lin et al. 2008; Menant et al. 2006), which, analogous to the situation for Gap1 (Ghaddar et al. 2014), is likely caused by substrate-induced conformational changes that promote the recognition of these permeases by arrestin-like adaptors of the Rsp5 ubiquitin ligase (Guiney et al. 2016; Hatakeyama et al. 2010; Nikko and Pelham 2009). In our experimental setting, cells were not exposed to methionine and only to rather low amounts of inositol (11 µM) in the medium, which is why both permeases were quite stably concentrated at the plasma membrane in exponentially growing cells. Conceivably, however, TORC1 inhibition may increase the cytoplasmic levels of these metabolites as a result of the inhibition of methionine- and inositol-consuming anabolic processes. If this were the case, it would be interesting to address the question whether high cytoplasmic levels of these or other metabolites can, similarly as proposed in the case of the transporter-related Ssy1 amino acid sensor (Wu et al. 2006), induce conformational changes in permeases, which then would trigger their endocytosis. Intriguingly, TORC1 downregulation may then, perhaps like treatment of cells with cycloheximide (Lin et al. 2008; Nikko and Pelham 2009), control permease sorting indirectly by causing specific metabolites to accumulate in the cytoplasm. Finally, TORC1 may also control MVB sorting by other means such as for instance by promoting the expression and/or stability of Ist1 that functions as an ESCRT inhibitor (Jones et al. 2012).

The v-ATPase acts upstream of TORC1 in flies, mammals, and yeast (Dechant et al. 2014; Eskes et al. 2018; Hatakeyama et al. 2019; Stauffer and Powers 2016; Zoncu et al. 2011). Our finding that TORC1 prevents microautophagic degradation of the v-ATPase subunit Vph1, therefore, indicates the existence of a positive feedback control mechanism that may contribute to the robustness of TORC1 signaling. The degradation of Vph1-GFP upon inhibition of TORC1, however, is rather slow and only affects a minor fraction of the total pool of Vph1-GFP over the period studied (which also explains why the 3-h rapamycin treatment does not visibly enhance the GFP signal within the vacuoles). This raises the question whether Vph1 is specifically targeted for degradation by a vacuolar membrane-resident ubiquitin ligase or whether, due to its abundance within the vacuolar membrane, follows the microautophagic degradation of other proteins. Thus, future progress in this area will not only require the analysis of the ubiquitinated residues within Vph1 (Swaney et al. 2013), but also the identification of additional bona fide microautophagic substrates. The phosphomimetic Vps27^7D^ allele that specifically compromises Vps27-dependent microautophagy will likely be useful in these efforts, which should ultimately clarify the importance of microautophagic processes for the survival of starved cells.

## References

[CR1] Albert V, Hall MN (2015). mTOR signaling in cellular and organismal energetics. Curr Opin Cell Biol.

[CR2] Beck T, Schmidt A, Hall MN (1999). Starvation induces vacuolar targeting and degradation of the tryptophan permease in yeast. J Cell Biol.

[CR3] Brachmann CB, Davies A, Cost GJ, Caputo E, Li J, Hieter P, Boeke JD (1998). Designer deletion strains derived from *Saccharomyces cerevisiae* S288C: a useful set of strains and plasmids for PCR-mediated gene disruption and other applications. Yeast.

[CR4] Brune T, Kunze-Schumacher H, Kolling R (2019). Interactions in the ESCRT-III network of the yeast *Saccharomyces cerevisiae*. Curr Genet.

[CR5] Dauner K, Eid W, Raghupathy R, Presley JF, Zha X (2017). mTOR complex 1 activity is required to maintain the canonical endocytic recycling pathway against lysosomal delivery. J Biol Chem.

[CR6] Dechant R, Saad S, Ibáñez AJ, Peter M (2014). Cytosolic pH regulates cell growth through distinct GTPases, Arf1 and Gtr1, to promote Ras/PKA and TORC1 activity. Mol Cell.

[CR7] Dobzinski N, Chuartzman SG, Kama R, Schuldiner M, Gerst JE (2015). Starvation-dependent regulation of golgi quality control links the TOR signaling and vacuolar protein sorting pathways. Cell Rep.

[CR8] Ejzykowicz DE, Locken KM, Ruiz FJ, Manandhar SP, Olson DK, Gharakhanian E (2017). Hygromycin B hypersensitive (*hhy*) mutants implicate an intact trans-Golgi and late endosome interface in efficient Tor1 vacuolar localization and TORC1 function. Curr Genet.

[CR9] Eltschinger S, Loewith R (2016). TOR complexes and the maintenance of cellular homeostasis. Trends Cell Biol.

[CR10] Eskes E, Deprez MA, Wilms T, Winderickx J (2018). pH homeostasis in yeast; the phosphate perspective. Curr Genet.

[CR11] Forgac M (2007). Vacuolar ATPases: rotary proton pumps in physiology and pathophysiology. Nat Rev Mol Cell Biol.

[CR12] Ghaddar K, Merhi A, Saliba E, Krammer EM, Prevost M, André B (2014). Substrate-induced ubiquitylation and endocytosis of yeast amino acid permeases. Mol Cell Biol.

[CR13] Guiney EL, Klecker T, Emr SD (2016). Identification of the endocytic sorting signal recognized by the Art1-Rsp5 ubiquitin ligase complex. Mol Biol Cell.

[CR14] Hatakeyama R, De Virgilio C (2019). A spatially and functionally distinct pool of TORC1 defines signaling endosomes in yeast. Autophagy.

[CR15] Hatakeyama R, Kamiya M, Takahara T, Maeda T (2010). Endocytosis of the aspartic acid/glutamic acid transporter Dip5 is triggered by substrate-dependent recruitment of the Rsp5 ubiquitin ligase via the arrestin-like protein Aly2. Mol Cell Biol.

[CR16] Hatakeyama R, Péli-Gulli MP, Hu Z, Jaquenoud M, Garcia Osuna GM, Sardu A, Dengjel J, De Virgilio C (2019). Spatially distinct pools of TORC1 balance protein homeostasis. Mol Cell.

[CR17] Henne WM, Buchkovich NJ, Emr SD (2011). The ESCRT pathway. Dev Cell.

[CR18] Jin N, Mao K, Jin Y, Tevzadze G, Kauffman EJ, Park S, Bridges D, Loewith R, Saltiel AR, Klionsky DJ, Weisman LS (2014). Roles for PI(3,5)P2 in nutrient sensing through TORC1. Mol Biol Cell.

[CR19] Jones CB, Ott EM, Keener JM, Curtiss M, Sandrin V, Babst M (2012). Regulation of membrane protein degradation by starvation-response pathways. Traffic.

[CR20] Kamada Y, Yoshino K, Kondo C, Kawamata T, Oshiro N, Yonezawa K, Ohsumi Y (2010). Tor directly controls the Atg1 kinase complex to regulate autophagy. Mol Cell Biol.

[CR21] Khmelinskii A, Keller PJ, Bartosik A, Meurer M, Barry JD, Mardin BR, Kaufmann A, Trautmann S, Wachsmuth M, Pereira G, Huber W, Schiebel E, Knop M (2012). Tandem fluorescent protein timers for in vivo analysis of protein dynamics. Nat Biotechnol.

[CR22] Lai K, Bolognese CP, Swift S, McGraw P (1995). Regulation of inositol transport in *Saccharomyces cerevisiae* involves inositol-induced changes in permease stability and endocytic degradation in the vacuole. J Biol Chem.

[CR23] Lang MJ, Martinez-Marquez JY, Prosser DC, Ganser LR, Buelto D, Wendland B, Duncan MC (2014). Glucose starvation inhibits autophagy via vacuolar hydrolysis and induces plasma membrane internalization by down-regulating recycling. J Biol Chem.

[CR24] Lin CH, MacGurn JA, Chu T, Stefan CJ, Emr SD (2008). Arrestin-related ubiquitin-ligase adaptors regulate endocytosis and protein turnover at the cell surface. Cell.

[CR25] MacGurn JA, Hsu PC, Smolka MB, Emr SD (2011). TORC1 regulates endocytosis via Npr1-mediated phosphoinhibition of a ubiquitin ligase adaptor. Cell.

[CR26] Mageswaran SK, Johnson NK, Odorizzi G, Babst M (2015). Constitutively active ESCRT-II suppresses the MVB-sorting phenotype of ESCRT-0 and ESCRT-I mutants. Mol Biol Cell.

[CR27] Marat AL, Haucke V (2016). Phosphatidylinositol 3-phosphates—at the interface between cell signalling and membrane traffic. EMBO J.

[CR28] Markgraf DF, Ahnert F, Arlt H, Mari M, Peplowska K, Epp N, Griffith J, Reggiori F, Ungermann C (2009). The CORVET subunit Vps8 cooperates with the Rab5 homolog Vps21 to induce clustering of late endosomal compartments. Mol Biol Cell.

[CR29] Menant A, Barbey R, Thomas D (2006). Substrate-mediated remodeling of methionine transport by multiple ubiquitin-dependent mechanisms in yeast cells. EMBO J.

[CR30] Merhi A, André B (2012). Internal amino acids promote Gap1 permease ubiquitylation via TORC1/Npr1/14-3-3-dependent control of the Bul arrestin-like adaptors. Mol Cell Biol.

[CR31] Mizushima N, Yoshimori T, Ohsumi Y (2011). The role of Atg proteins in autophagosome formation. Annu Rev Cell Dev Biol.

[CR32] Müller M, Schmidt O, Angelova M, Faserl K, Weys S, Kremser L, Pfaffenwimmer T, Dalik T, Kraft C, Trajanoski Z, Lindner H, Teis D (2015). The coordinated action of the MVB pathway and autophagy ensures cell survival during starvation. eLife.

[CR33] Nikko E, Pelham HR (2009). Arrestin-mediated endocytosis of yeast plasma membrane transporters. Traffic.

[CR34] Odorizzi G, Babst M, Emr SD (1998). Fab1p PtdIns(3)P 5-kinase function essential for protein sorting in the multivesicular body. Cell.

[CR35] Oku M, Sakai Y (2018). Three distinct types of microautophagy based on membrane dynamics and molecular machineries. BioEssays.

[CR36] Oku M, Maeda Y, Kagohashi Y, Kondo T, Yamada M, Fujimoto T, Sakai Y (2017). Evidence for ESCRT- and clathrin-dependent microautophagy. J Cell Biol.

[CR37] Rahman MA, Terasawa M, Mostofa MG, Ushimaru T (2018). The TORC1-Nem1/Spo7-Pah1/lipin axis regulates microautophagy induction in budding yeast. Biochem Biophys Res Commun.

[CR38] Raymond CK, Howald-Stevenson I, Vater CA, Stevens TH (1992). Morphological classification of the yeast vacuolar protein sorting mutants: evidence for a prevacuolar compartment in class E *vps* mutants. Mol Biol Cell.

[CR39] Rieder SE, Banta LM, Kohrer K, McCaffery JM, Emr SD (1996). Multilamellar endosome-like compartment accumulates in the yeast *vps28* vacuolar protein sorting mutant. Mol Biol Cell.

[CR40] Rothman JH (1989). Acidification of the lysosome-like vacuole and the vacuolar H^+^-ATPase are deficient in two yeast mutants that fail to sort vacuolar proteins. J Cell Biol.

[CR41] Saxton RA, Sabatini DM (2017). mTOR signaling in growth, metabolism, and disease. Cell.

[CR42] Schmidt A, Beck T, Koller A, Kunz J, Hall MN (1998). The TOR nutrient signalling pathway phosphorylates NPR1 and inhibits turnover of the tryptophan permease. EMBO J.

[CR43] Sikorski RS, Hieter P (1989). A system of shuttle vectors and yeast host strains designed for efficient manipulation of DNA in *Saccharomyces cerevisiae*. Genetics.

[CR44] Stauffer B, Powers T (2016). Target of rapamycin signaling mediates vacuolar fragmentation. Curr Genet.

[CR45] Suzuki K, Akioka M, Kondo-Kakuta C, Yamamoto H, Ohsumi Y (2013). Fine mapping of autophagy-related proteins during autophagosome formation in *Saccharomyces cerevisiae*. J Cell Sci.

[CR46] Swaney DL, Beltrao P, Starita L, Guo A, Rush J, Fields S, Krogan NJ, Villen J (2013). Global analysis of phosphorylation and ubiquitylation cross-talk in protein degradation. Nat Methods.

[CR47] Urban J, Soulard A, Huber A, Lippman S, Mukhopadhyay D, Deloche O, Wanke V, Anrather D, Ammerer G, Riezman H, Broach JR, De Virgilio C, Hall MN, Loewith R (2007). Sch9 is a major target of TORC1 in *Saccharomyces cerevisiae*. Mol Cell.

[CR48] Wen X, Klionsky DJ (2016). An overview of macroautophagy in yeast. J Mol Biol.

[CR49] Wu B, Ottow K, Poulsen P, Gaber RF, Albers E, Kielland-Brandt MC (2006). Competitive intra- and extracellular nutrient sensing by the transporter homologue Ssy1p. J Cell Biol.

[CR50] Zhu L, Jorgensen JR, Li M, Chuang YS, Emr SD (2017). ESCRTs function directly on the lysosome membrane to downregulate ubiquitinated lysosomal membrane proteins. eLife.

[CR51] Zoncu R, Bar-Peled L, Efeyan A, Wang S, Sancak Y, Sabatini DM (2011). mTORC1 senses lysosomal amino acids through an inside-out mechanism that requires the vacuolar H^+^-ATPase. Science.

